# Green synthesized silver nanoparticles using *Cyperus rotundus* L. extract as a potential antiviral agent against infectious laryngotracheitis and infectious bronchitis viruses in chickens

**DOI:** 10.1186/s40538-022-00325-z

**Published:** 2022-08-12

**Authors:** Zahraa Hamdi Abo-El-Yazid, Osama Konsowa Ahmed, Mohamed El-Tholoth, Mohamed Abdel-Shakur Ali

**Affiliations:** 1 Biochemistry Department, Faculty of Agriculture, Cairo University, Giza, Egypt; 2 Department of Virology, Faculty of Veterinary Medicine, Mansoura University, Mansoura, 35516 Egypt; 3 Health Sciences Division, Higher Colleges of Technology, Al Ain Men’s Campus, 17155 Al Ain, United Arab Emirates

**Keywords:** *Cyperus rotundus*, Chickens, Infectious laryngotracheitis virus, Infectious bronchitis virus, Avian coronavirus, Silver nanoparticles, Antiviral activity

## Abstract

**Background:**

Infectious laryngotracheitis (ILT) and infectious bronchitis (IB) are two common respiratory diseases of poultry that inflict great economic burden on the poultry industry. Developing an effective agent against both viruses is a crucial step to decrease the economic losses. Therefore, for the first time green synthesized silver nanoparticles using *Cyperus rotundus* L*.* aqueous extract was evaluated in vitro as a potential antiviral against both viruses.

**Results:**

Silver nanoparticles from *Cyperus rotundus* were characterized by the spherical shape, 11–19 nm size, and zeta potential of − 6.04 mV. The maximum nontoxic concentration (MNTC) was 50 µg mL^−1^ for both viruses without harmful toxicity impact. The study suggested that some of the compounds in *C. rotundus* extract (gallic acid, chlorogenic acid, and naringenin) or its silver nanoparticles could interact with the external envelope proteins of both viruses, and inhibiting extracellular viruses.

**Conclusions:**

The results highlight that *C. rotundus* green synthesized silver nanoparticles could have antiviral activity against infectious laryngotracheitis virus (ILTV) and infectious bronchitis virus (IBV) in chickens.

**Graphical Abstract:**

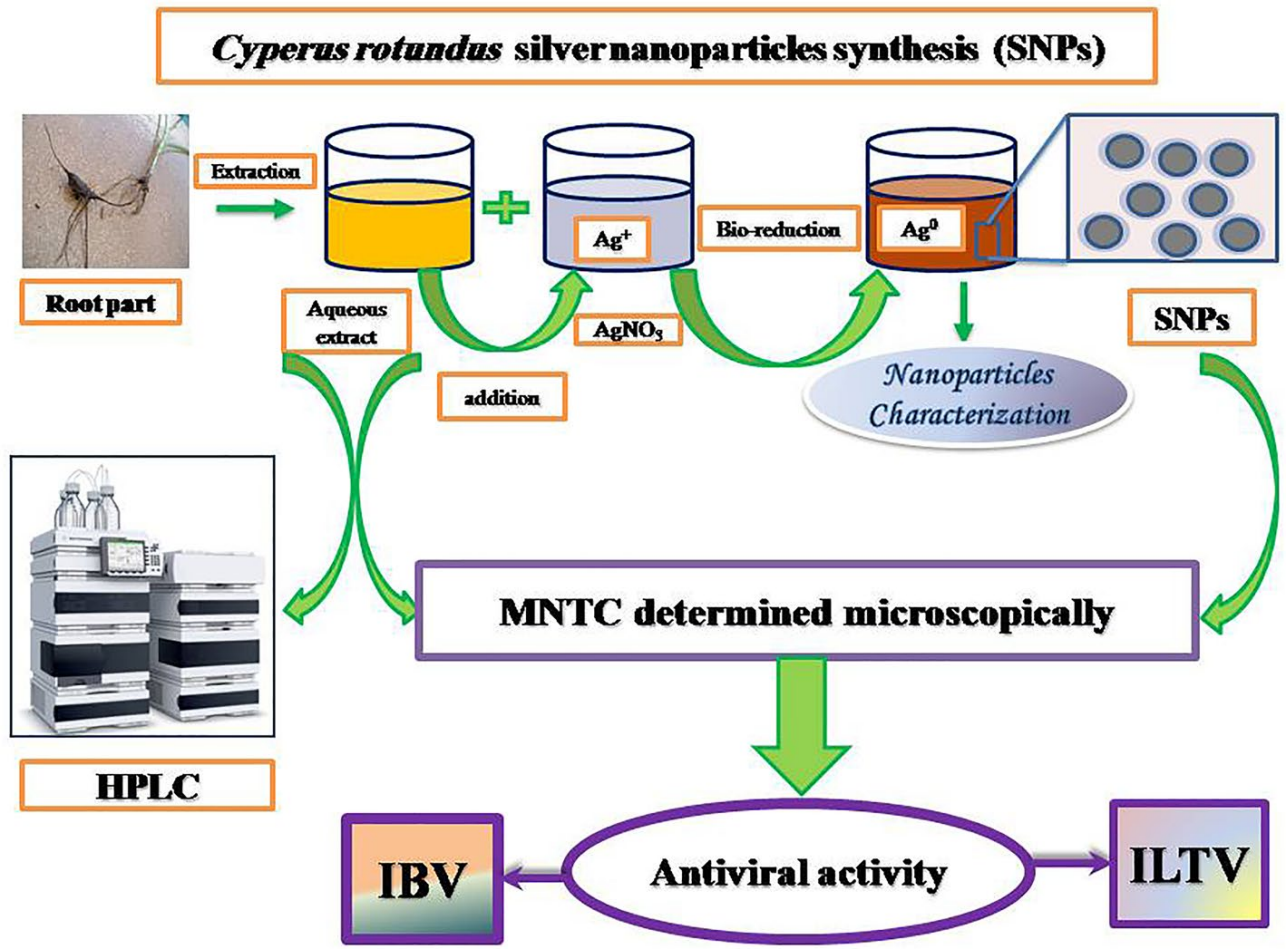

## Background

*Cyperus rotundus* Linn. (Family *Cyperaceae*), sometimes called the coco-grass, Java grass, nutgrass, or purple nutsedge is labeled as the world's worst weed that can damage the ecosystem and has strong invasive nature. The plant has beneficial pharmacological, and therapeutic activities like antioxidant property, anti-inflammatory activity, antimicrobial activity, hepatoprotective activity, cytoprotective effect, antiviral activity, and anti-cariogenic property. It has different secondary metabolites including phenolic acid, flavonoids, alkaloids, iridoids, tannins, glycosides, saponins, terpenoids, and some isolated phenolics [1–6].

*Cyperus rotundus* Linn. was used in India, West Asia, ancient Egypt and Chinese traditional medicine for the treatment of various human diseases like bronchitis, stomach ache, liver diseases, disorders of menstrual cycles, leprosy, diarrhea, fever, parasitic infestation and renal colic [7–9]. There are no reports on usage of such plant in the veterinary field to treat infection with viruses infecting respiratory tract of chickens as infectious laryngotracheitis (ILT) and infectious bronchitis (IB) viruses.

ILT is caused by a double-stranded DNA virus named *gallid alphaherpesvirus* 1 (GaHV-1), commonly known as infectious laryngotracheitis virus (ILTV) that belongs to the *Alphaherpesvirinae* subfamily within the *Herpesviridae* family. IB is caused by a single-stranded RNA Gammacoronavirus. Both diseases share similar symptoms that include respiratory distress, high mortality rates, poor feed conversion rates, drop in egg production, and susceptibility to other respiratory tract infections [10–12]**.** Therefore, they impose huge economic burden on the industry. Both causative viruses (herpesvirus and coronavirus) are enveloped with different structural viral proteins incorporated in the virus envelope [13, 14].

Vaccination is the major way of controlling both ILT and IB [13, 14]. Live attenuated and killed virus vaccines are used for chicken’s protection against both diseases. However, limitations facing usage of live-attenuated vaccines for controlling include: reversion to virulence, recombination between vaccine and field strains that may develop new virus variants and the potential effect of maternal antibody on vaccine efficacy [15–17]. On the other hand, killed vaccines require priming with live-attenuated vaccines and multiple vaccinations due to the short duration of induced immunity [18, 19]. Moreover, there have been reports of ILT and IB outbreaks in Egypt although chickens were vaccinated [20, 21]. Therefore, developing of new alternative and complementary strategies that target different variants of viruses is warranted. One of these strategies could be the use of green synthesized nanoparticles using various plants as antiviral agents.

Green synthesis of silver nanoparticles (SNPs) using various plants constituents is environment-friendly, nonpathogenic, and cost-effective way for medicinal applications, providing nontoxic, and safe reagents. Interestingly, SNPs has been reported to reveal antimicrobial and catalytic antiviral activities. *C. rotundus* extract is used as reducing agent for silver metal ions, and responsible for stabilizing and capping process for SNPs. Therefore, phytochemical classes contribute to the green synthesis of *C. rotundus* silver nanoparticle; The SNPs prevent viral replication by binding to the viral/host proteins and/or nucleic acid [4, 22–27].

The current research was conducted to study for the first time the inhibitory effect of green synthesized silver *C. rotundus* nanoparticles as an antiviral agent against ILT and IB viruses and determine active compounds that are responsible for such antiviral activity.

## Results and discussion

### Phenolics and flavonoids profile in *C. rotundus* aqueous extract by high-performance liquid chromatography (HPLC)

Table 1 depicts the 14 phenolic and flavonoid compounds detected in *C. rotundus* aqueous extract by the HPLC technique. Some of these compounds had antiviral activity as previously reported [28–37], such as gallic acid, chlorogenic acid, naringenin, rutin, kaempferol, methyl gallate, and quercetin with concentrations of 9259.23, 1434.62, 762.60, 87.03, 65.04, 54.80, and 18.60 µg g^−1^, respectively, and percentages of 45.30, 13.67, 10.86, 0.59, 1, 3.22, and 0.21%, respectively. Gallic acid, chlorogenic acid, and naringenin were the major compounds in the HPLC profile of *C. rotundus* aqueous extract. They had various activities like antioxidant, anti-inflammatory, antibacterial, antiviral, antimutagenic and anticancer activity.

**Table 1:** HPLC analysis of phenolic and flavonoid compounds in *Cyperus rotundus* aqueous extract

No.	Compounds’ name	Area sum %	Concentrations (µg g^−1^)	Type
1	Gallic acid	45.30	9259.23	P
2	Chlorogenic acid	13.67	1434.62	P
3	Naringenin	10.86	762.60	F
4	Ellagic acid	2.70	357.28	P
5	Ferulic acid	4.91	264.90	P
6	Syringic acid	4.03	261.66	P
7	Coumaric acid	10.35	225.34	P
8	Catechin	0.79	114.50	F
9	Rutin	0.59	87.03	F
10	Kaempferol	1.00	65.04	F
11	Methyl gallate	3.22	54.80	P
12	Vanillin	1.86	53.90	P
13	Quercetin	0.21	18.60	F
14	Cinnamic acid	0.43	5.84	P
15	Caffeic acid	ND	ND	P
16	Pyrocatechol	ND	ND	P
17	Hesperetin	ND	ND	F

P, phenolic compound; F, flavonoid compound; ND, not determined

Gallic acid, chlorogenic acid, and naringenin act as antioxidants by scavenging free radicals and reducing lipid peroxidation-mediated oxidative DNA damage. These compounds' hydroxyl substituents (OH) are responsible for their antioxidant action. High reactivity against reactive oxygen species (ROS) and reactive nitrogen species (RNS) is exhibited by these hydroxyl groups. The reported virucidal activity of the mentioned compounds may be as a result of the hydrophobic interaction between the hydroxyl group and the components of virion and/or the inhibition of the life cycle of viruses by either targeting viral envelopes or viral replication enzymes [30, 38–40].

### *C. rotundus* SNPs characterization

#### UV–VIS spectrum of *C. rotundus* SNPs

**Fig. 1:**
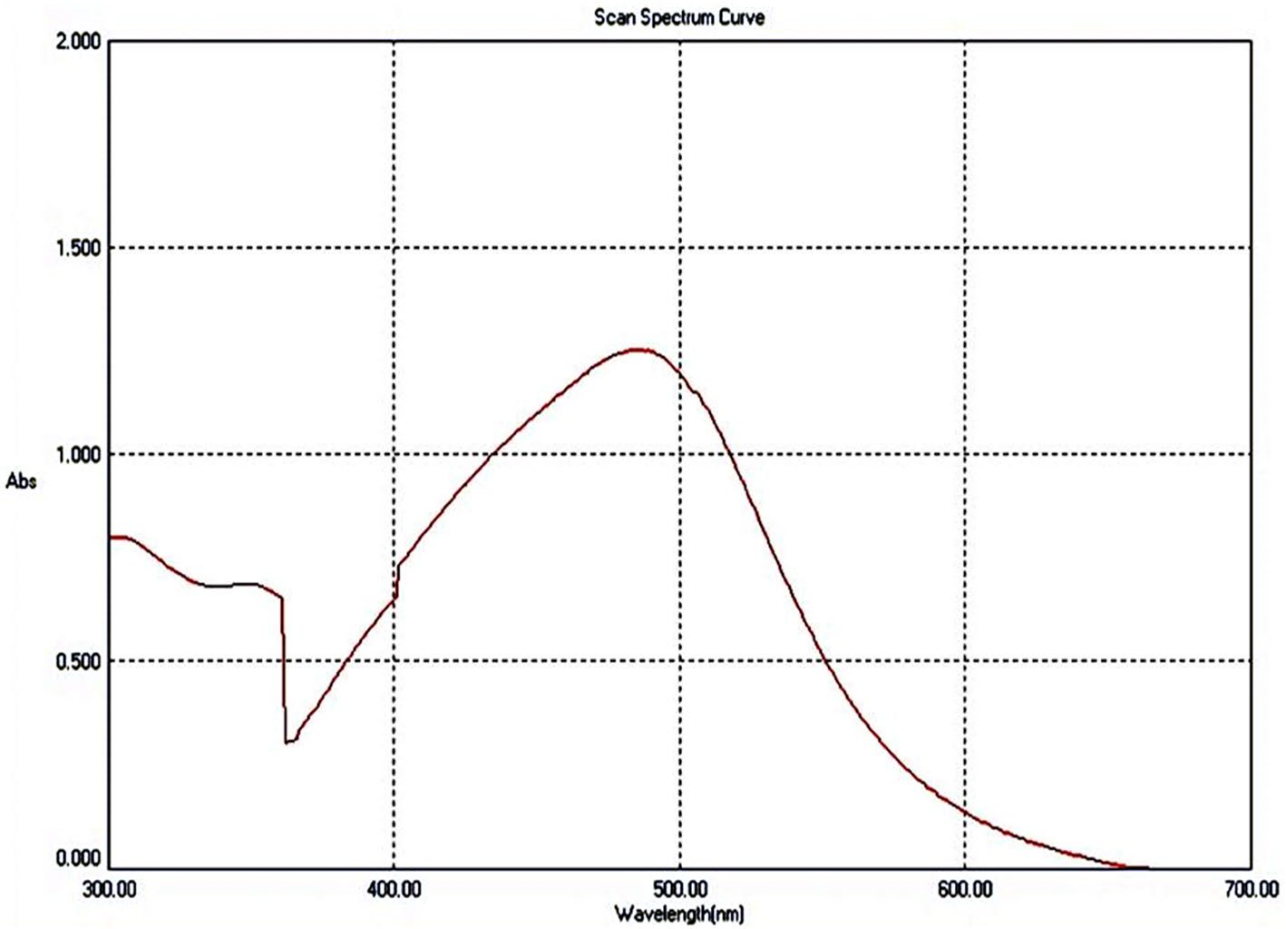
UV–visible spectrum of *Cyperus rotundus* silver nanoparticles

Figure 1 depicts the UV–VIS spectrum of the synthesized SNPs. SNPs had a single maximum absorbance located at the lower wavelength. The maximum peaks at 484 nm, suggesting that the SNPs were formed by absorbance at 1.25 after incubation period in dark overnight at room temperature [4].

**Fig. 2:**
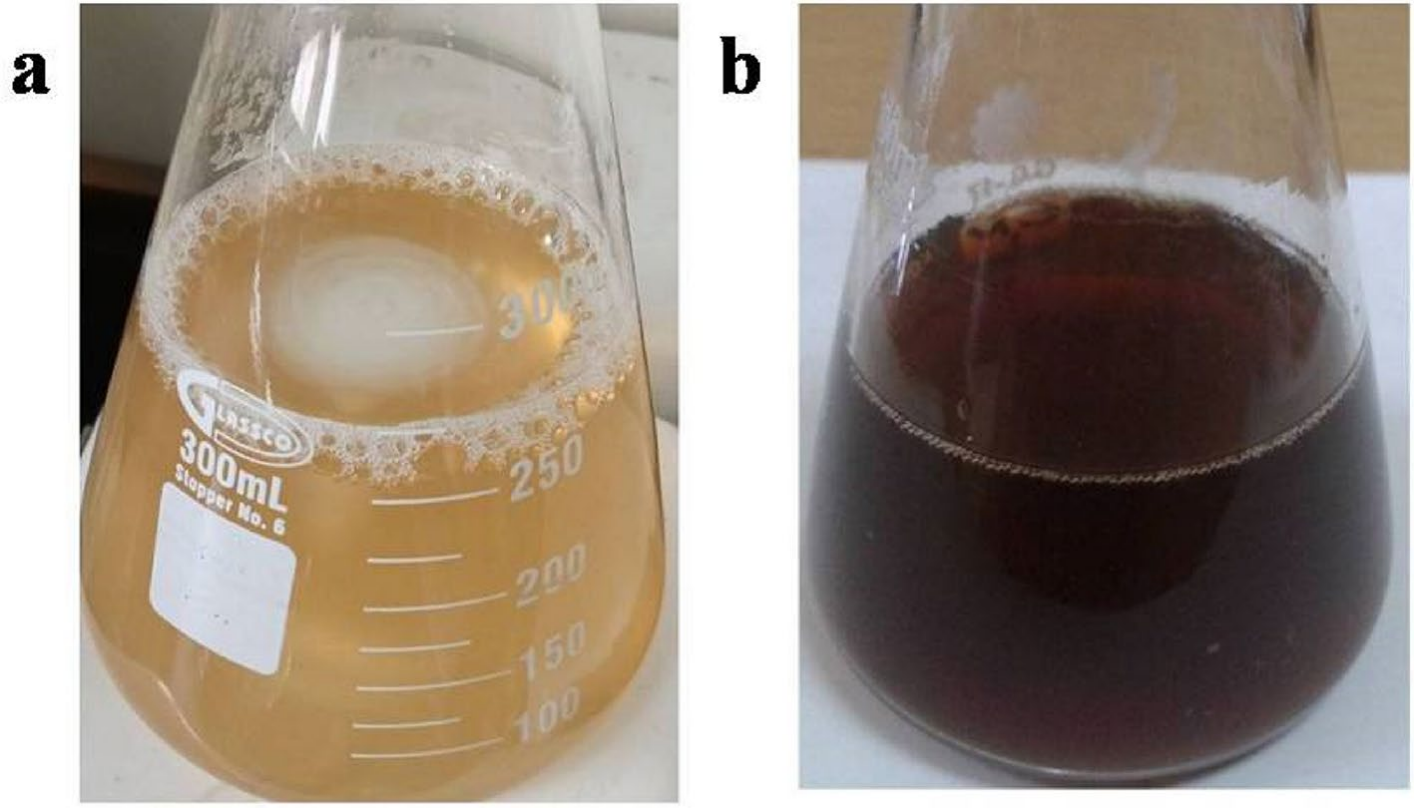
*Cyperus rotundus* aqueous extract morphology change in color

The bio-reduction of silver ion to the nanoparticles via biochemical contents presented within the aqueous extract in solution color conversion from slightly yellow to heavy reddish as in Fig. 2. In the green biosynthesis, AgNO_3_ contained Ag^+^ and was bio-reduced to silver Ag^0^, forming the nanoparticle silver core due to surface plasmon vibration excitation in SNPs which have free electrons and give rise it to a surface plasmon resonance (SPR) absorption [41].

#### Transmission electron microscopy (TEM) of *C. rotundus* SNPs

**Fig. 3:**
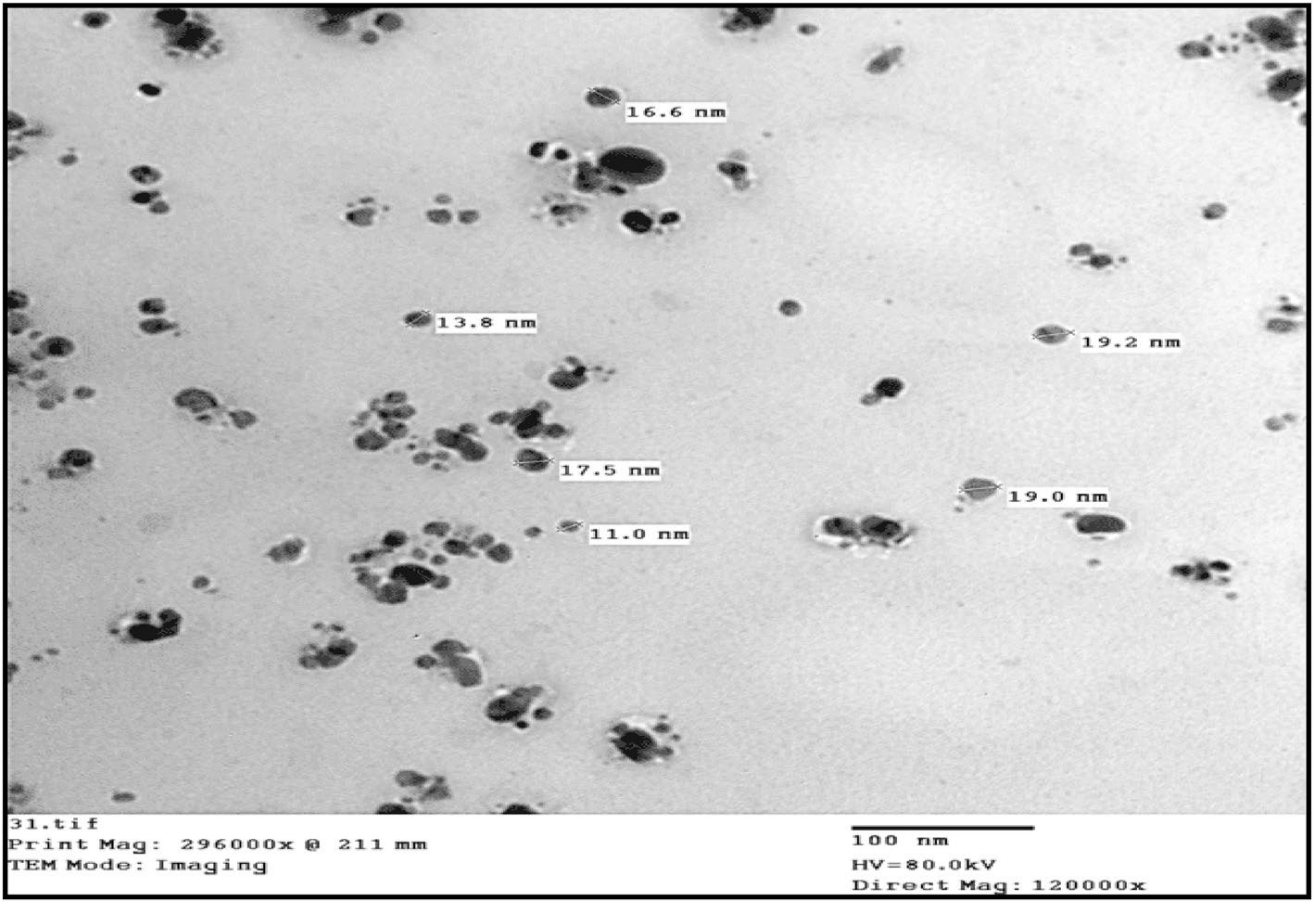
Transmission electron microscope micrograph of *Cyperus rotundus* silver nanoparticles

The TEM micrographs (Fig. 3) illustrated the morphology and size of the SNP of *C. rotundus* which are spherical in shape with size ranging from 11 to 19 nm, which agreed with the SPR of UV–VIS Spectrum in our results [42, 43].

#### Zeta potential and particle size of *C. rotundus* SNPs

**Fig. 4:**
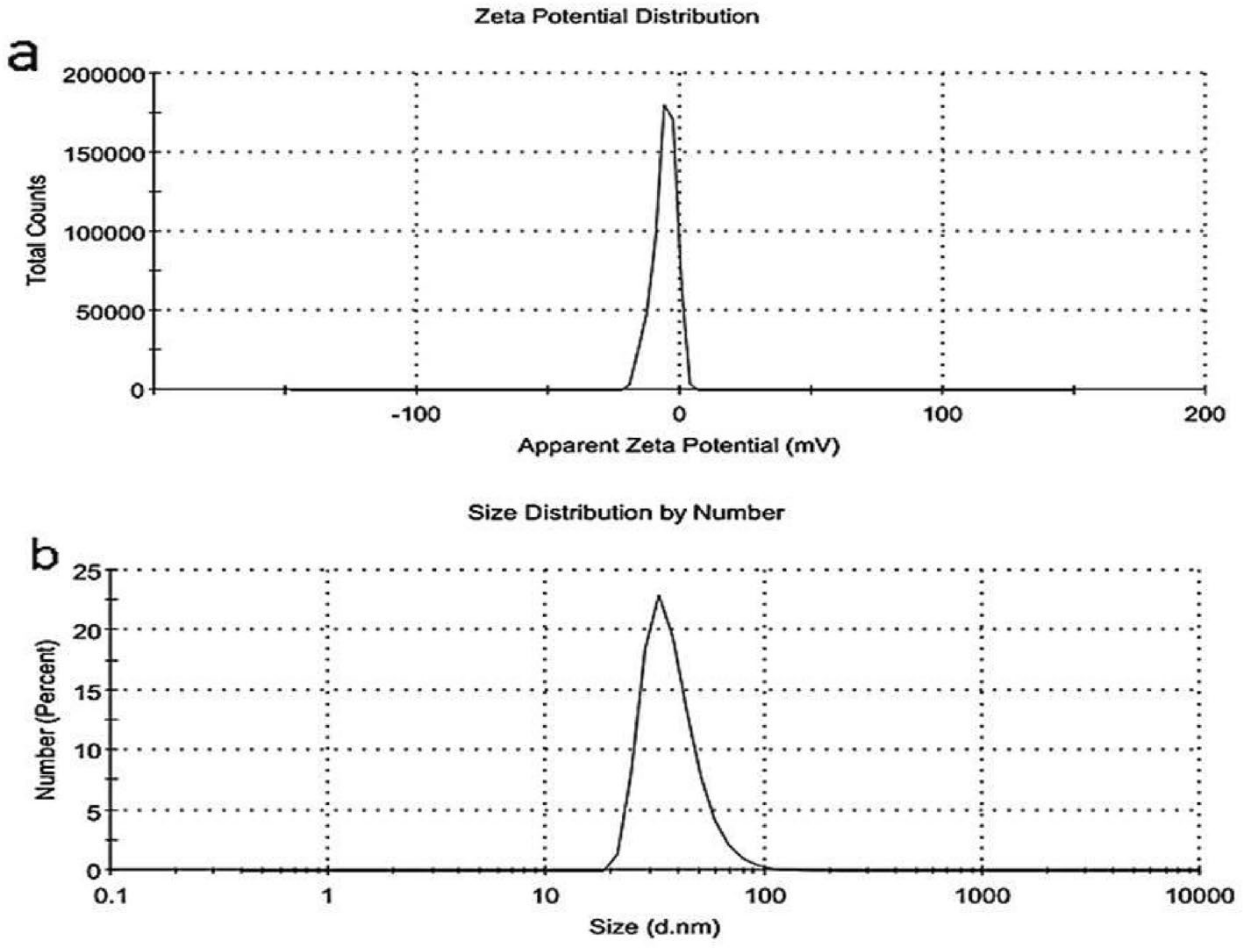
Zeta potential (**a**) and DLS (**b**) of *Cyperus rotundus* SNPs

As shown in Fig. 4a the zeta potential value of *C. rotundus* SNPs was negatively charged value (− 6.04 mV) which could affect cellular, viral proteins, and/or interaction with each other. On the other hand, the average size of *C. rotundus* SNPs was 38.06 nm, and it has only one sharp peak which means good biosynthesis of SNPs (Fig. 4b).

#### Attenuated total reflectance-Fourier transform-infrared spectrum (ATR-FT-IR) of *C. rotundus* SNPs

**Fig. 5:**
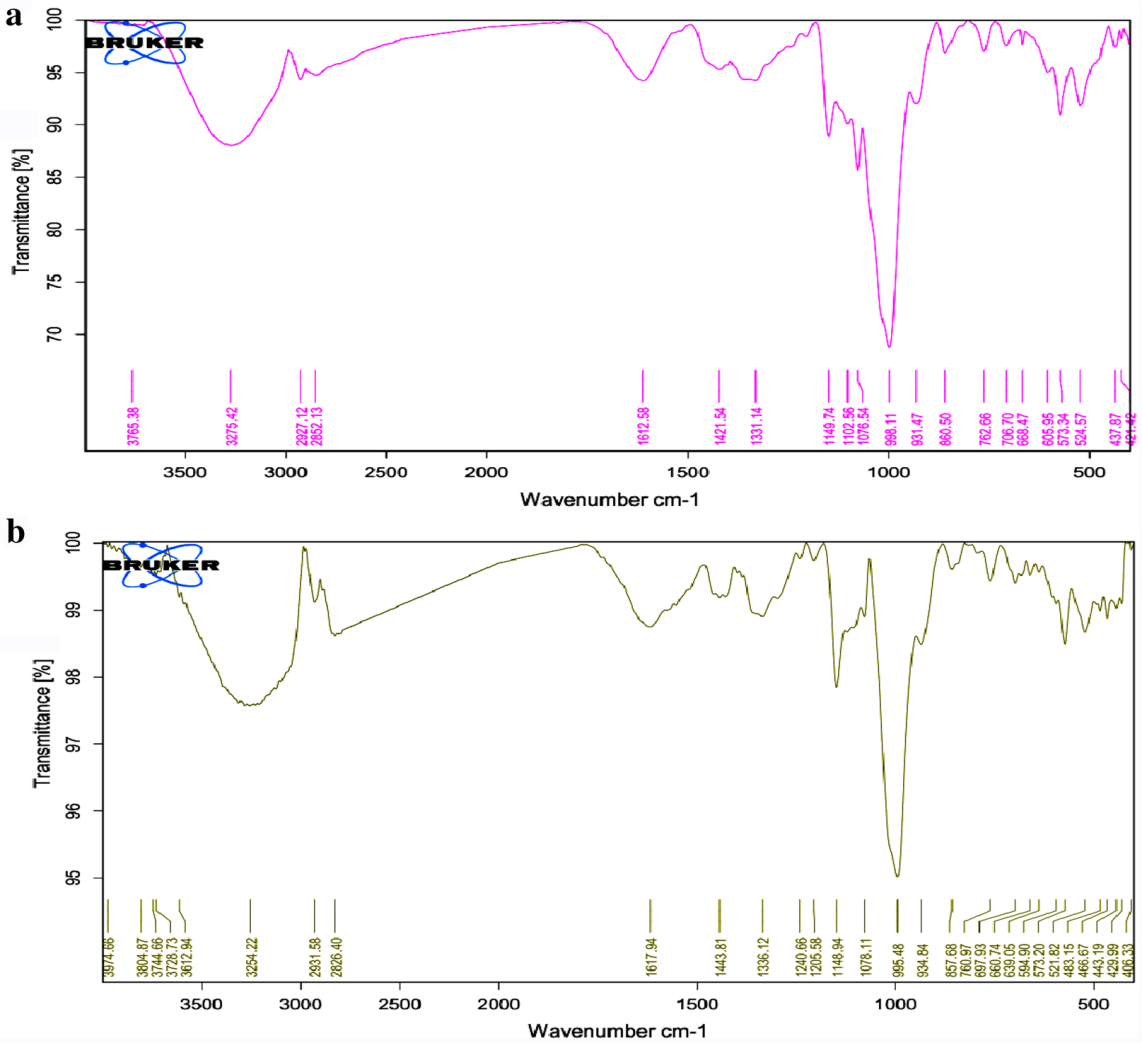
ATR-FT-IR spectrum of *Cyperus rotundus* silver nanoparticles

The SNPs ATR-FT-IR spectrum (Fig. 5b) revealed absorption peaks at 3254 cm^−1^ due to –O–H stretching vibration, while those at 2923 and 2853 cm^−1^ due to C–H stretching vibration. The presence of biomolecules in *C. rotundus* extract (Fig. 5a) can be assigned from the peak at 1612 cm^−1^ became 1617 cm^−1^of C=O stretching vibration as well as C–C bond stretching and C–O–H bending vibrations at 1331 and 1076 cm^−1^ became 1336 cm^−1^ and 1078 cm^−1^, respectively.

In addition, C–O–C stretching vibration for SNPs at 1148, 1205 and 1240 cm^−1^ and 1149 cm^−1^ for *C. rotundus* extracts and aromatic C–H in-plane bending at 998 cm^−1^ for aqueous extract became 995 cm^−1^ in SNPs. The appearance of peaks at 594 cm^−1^ showed Ag–O binding in SNPs [22].

The SNPs formation in presence of phenolic compounds which had aromatic ring act as high nucleophilic agent attached to free hydroxyl groups and carbonyl groups (gallic acid, chlorogenic acid, ferulic acid, etc.) are able to bind and reduce the metal salt into a nanoparticle form.

Also in case of flavonoid compounds (naringenin, rutin, quercetin, kaempferol) which contain the oligosaccharide moiety has multi-hydroxyl groups bind with aglycone part in glycoside leading to enol–keto tautomerism and reactive hydrogen atom to bio-reduction process [4, 44]. From previous results, *C. rotundus* aqueous extract not only used for reducing metal salt and the core formation of SNPs, but also act as stabilizing agent for SNPs.

### Cytotoxicity effect

The cytotoxicity of aqueous extract and green synthesized SNPs were tested on the Vero cells by MNTC method of each extract that determined in the range of the cytotoxic concentrations at 0–600 µg mL^−1^ for aqueous extract, and 0–200 µg mL^−1^for SNPs pre- and post-infection treatment using 10^4.8^ and 10^3.7^TCID_50_ mL^−1^ for IBV and ILTV viruses, respectively.

Microscopically demonstrated normal cell morphology was observed and no cytotoxic effect of the extracts on the Vero cells in the range of the cytotoxic concentrations at 0–400 µg mL^−1^ of aqueous extract, and 0–50 µg mL^−1^ for SNPs (Table 2) [45, 46].

**Table 2:** MNTC^a^ and IC_50_^b^ for *C. rotundus* extracts against ILTV and IBV

Extract name	MNTC of Vero cells (µg mL^−1^)	IC_50_ ILTV (µg mL^−1^)	IC_50_ IBV (µg mL^−1^)
Aqueous	≤ 400	310	ND
SNP pre-infection	≤ 50	16	19
SNP post-infection	≤ 50	9.5	9.5

*ND* not determined

^a^Maximum nontoxic concentration

^b^The 50% inhibitory concentrations of infected Vero cells

### Assessment of antiviral activity

The antiviral activity was measured against ILTV and IBV using *C. rotundus* aqueous extract and expressed as infected cell percentage (Table 3). In the case of anti-ILTV activity, there was significantly different at 400 µg mL^−1^ (IC_50_ 310 µg mL^−1^) which had the lowest infection percentage (41.07 ± 0.9%), while the anti-IBV activity was not efficient and showed infection percentage of 95.20 ± 0.20% in comparison to the virus-infected controls.

**Table 3:** Anti-ILTV and IBV activity of *C. rotundus* aqueous extract at different concentrations

Concentrations (µg mL^−1^)	ILTV-infected Vero cells %	IBV-infected Vero cells %
0	100.0 ± 0.00^a^	100.0 ± 0.00^a^
150	72.17 ± 11.60^b^	98.60 ± 0.53^b^
250	55.75 ± 5.05^c^	91.06 ± 1.05^d^
400	41.07 ± 0.90^d^	95.20 ± 0.20^c^

Each value represents the mean ± standard deviation, *n* = 3. Values within columns that are followed by the same letter do not differ significantly at *p* < 0.01

Gallic acid, naringenin, and chlorogenic acid were predicted to have anti-ILTV (*gallid alphaherpesvirus* 1) activity since they demonstrated high anti-herpetic efficacy [31–37]. They in particular, had 22-, 13.8-, and 3.5-times higher concentrations than previously reported [28, 29, 37]. Gallic acid has a virucidal activity against herpes because it has an aromatic structure that can inhibit viral protein expression [30].

The polyphenolic compounds, both flavonoids, and phenolic acids which had virucidal activity because they have a high binding affinity to viral and/or host cell membrane protein and form a complex that prevent absorption of the virus in the early stage of the herpes virus replication cycle [28]. Results in Table 1 indicate that these polyphenolic compounds may have the potential to bind to ILTV envelope glycoproteins and form a complex that inhibits the virus' ability to infect host cells, as shown in Table 3 that 400 µg mL^−1^ concentration has 41.07% ILTV-infected cells.

The results in Tables 4 and 5 exhibited the ILTV and IBV-infected Vero cell percentage following pre- and post-infection treatment with *C. rotundus* SNPs. The infection percentages at concentration of 50 µg mL^−1^ for pre- and post-infection treatment were 27.35 ± 0.75%, and 14.0 ± 4.2%, respectively, for ILTV-infected cells while for IBV-infected cells were 29.10 ± 0.40 and 12.93 ± 0.80, respectively (IC_50_ 9.5 µg mL^−1^).

**Table 4:** Anti-ILTV activity of *C. rotundus* silver nanoparticles pre/post-infection at different concentrations

Concentrations (µg mL^−1^)	ILTV-infected Vero cells %	
	SNP treatment pre-infection	SNP treatment post-infection
0	100.0 ± 0.00^a^	100.0 ± 0.00^a^
12.5	54.35 ± 1.45^b^	31.73 ± 0.83^b^
25	37.80 ± 2.40^c^	19.03 ± 1.45^c^
50	27.35 ± 0.75^d^	14.00 ± 4.20^d^

Each value represents the mean ± standard deviation, *n* = 3. Values within columns that are followed by the same letter do not differ significantly at *p* < 0.01

**Table 5:** Anti-IBV activity of *C. rotundus* silver nanoparticles pre/post-infection at different concentrations

Concentrations (µg mL^−1^)	IBV-infected Vero cells %	
	SNP treatment pre-infection	SNP treatment post-infection
0	100.0 ± 0.00^a^	100.0 ± 0.00^a^
12.5	57.80 ± 2.60^b^	33.17 ± 2.06^b^
25	40.40 ± 0.90^c^	20.17 ± 4.95^c^
50	29.10 ± 0.40^d^	12.93 ± 0.80^d^

Each value represents the mean ± standard deviation, *n* = 3. Values within columns that are followed by the same letter do not differ significantly at *p* < 0.01

These results revealed significant antiviral activity of *C. rotundus* SNPs in both pre-infection and post-infection exposures. However, the efficacy of antiviral activity was increased when the infected cells were post-treated with SNPs.

Multiple plants have natural compounds which can be used for biologically reducing sliver salt to form SNPs via green synthesis; SNPs have many therapeutic effects, such as antiviral activity. Previous studies on HSV-1 revealed that SNPs of 4–13 nm have 80% antiviral activity [42, 43]. SNPs could bind to the disulfide link of gp120 receptor, inhibit hepatitis B virions (HBV) production in vitro, damage enveloped viral glycoproteins, block HSV-1 entry to host cell, as well as could react with G protein of respiratory syncytia virus (RSV) inhibiting specific virus attachment [47].

As described previously in [48], suppression of ILTV and IBV replication by SNPs could be explained by possible interaction of SNPs with enveloped glycoproteins' ILTV, such as gB. The pervious results demonstrated that multiple sequence alignment of gB ILTV with 6 other *Herpesviridae* members stated that both of 10 cysteine residues and N-linked glycosylation positions were completely and largely conserved, respectively. Therefore, they could react with host cell membrane glycoproteins' and inhibit the ILTV entry process [48]. Similarly, SNPs may interact in the same manner with spike proteins of the enveloped IBV resulting in significant anti-IBV efficacy. Moreover, SNPs are able to release silver ions that bind with thiol groups by the irreversible reaction within proteins such as respiratory enzymes in host cells and causing protein dysfunction. Also, they could react with nucleic acids (DNA and/or RNA) causing blockage of cellular pathways and /or viral replication cycle [49–52].

## Material and methods

### Aqueous extract preparation of *Cyperus rotundus*

The root part of *C. rotundus* was kindly authenticated in Flora and Phytotaxonomy Researches Department, Agriculture Museum, Egypt.

The root part was washed with tap water, dried, and ground into plant powder, and hot extracted at 80–90 °C for 30 min with stirring, then centrifuged at 2150×*g* and the filtrate collected and lyophilized, ready for use [53].

### Phenolics and flavonoids profile in *C. rotundus* aqueous extract by high-performance liquid chromatography (HPLC)

HPLC technique analysis was applied using an Agilent Technologies 1260 series. The separation has occurred via column type, ZORBAX Eclipse C_18_ (4.6 mm × 250 mm i.d., 5 μm as particle size). The mobile phase consisted of H_2_O (A) and acetonitrile containing 0.05% trifluoroacetic acid (B) at a flow rate of 1 mL/min. The mobile phase composition was programmed consecutively in a linear gradient as follows: 0 min (18% B); from 0 to 5 min (20% B); from 5 to 8 min (40% B); from 8 to 12 min (40% B); from 12 to 15 min (18% B); from 15 to 16 min (18%B) and from 16 to 20 (18%B). The multi-wavelength detector was monitored at 280 nm. The injection volume was 5 μL; the column temperature was 40 °C [54].

### Green synthesis of silver nanoparticles (SNPs)

A 5 mL of *C. rotundus* aqueous extract was added to 50 mL of prepared 1 mM silver nitrate (AgNO_3_) solution and stirred for 30 min. The purified nanoparticles were separated by centrifugation in a Beckman Coulter’s Avanti J-E centrifuge (USA) at 20,426×*g* for 20 min. [53].

### SNPs characterization

Physical properties, morphology and size of the prepared SNPs were characterized by various techniques. The ultraviolet–visible (UV–Vis) spectrum of synthesized SNPs was recorded using a UV–Vis spectrophotometer (PG Instruments Ltd T70). Attenuated total reflectance-Fourier transform infrared spectroscopy (BRUKER Vertex 80 V spectrophotometer, Germany, ATR-FTIR) was performed to detect the bio-functional groups present in the *C. rotundus* aqueous extract that may account for nanoparticles reduction and stabilization. Transmission electron microscopy (TEM; JOEL-JEM-1400TEM, Japan) analyzed the size and morphology of the nanoparticles. The size distribution of the synthesized SNPs was determined by dynamic light scattering (DLS) with a Zeta sizer Nano ZS (Malvern, UK) instrument at 25 °C.

### Cytotoxicity and antiviral activity

#### Cells and viruses

Vero cell line (African green monkey kidney cell line) was propagated in Dulbecco’s modified Eagle’s medium (DMEM; Gibco BRL, Grand Island, NY, USA) supplemented with 10% fetal bovine serum (FBS) (Invitrogen, Grand Island, MI), 100 U penicillin mL^−1^ (Gibco, Invitrogen), 0.1 mg streptomycin mL^−1^ (Invitrogen, Gaithersburg, MD, USA), 0.1 mg gentamycin mL^−1^ (Invitrogen, Gaithersburg, MD, USA), and 1% non-essential amino acids 100x (Invitrogen, Gaithersburg, MD, USA) and incubated at 37 °C with CO_2_ (5%). Cells were examined daily for confluency and sub-cultured when reached 90% confluency. The fifth passage was used for antiviral assay. Infectious laryngotracheitis virus (ILTV) and infectious bronchitis virus (IBV) were isolated originally from a disease outbreak in Dakahlyia Governorate, Egypt, and molecularly identified as described previously [55, 56].

#### Cytotoxicity effect

Cell suspensions were seeded at 100 µL/well of 96-well plates at a density of 10^5^ cells/mL. The cells were pre-incubated at 37 °C as stabilizations prior to the addition 100 µL of extract at ranged concentrations from 100 to 600 μg mL^−1^ for aqueous extract and from 12.5 to 200 μg mL^−1^ for SNP extract. Viability of the cells was assessed by ethidium monoazide bromide (EMA) staining, ensuring > 99% viability at the highest concentration of each extract. MNTC was evaluated by the microscopically observation of cells' morphological changes at 12 h of incubation [43].

#### In vitro infection experiments for assessment of antiviral activity

Antiviral activity experiments were conducted as previously described [57]. Vero cells were treated pre- and post-infection with various concentrations of *Cyperus rotundus* L. aqueous extract (150 to 400 μg mL^−1^) and corresponding SNPs (12.5 to 50 μg mL^−1^). For pre-infection exposure, 10^4.8^ and 10^3.7^ TCID_50_/mL for IBV and ILTV viruses, respectively were incubated at 37 °C with different *Cyperus rotundus* L. aqueous extract (150, 250, 400 µg mL^−1^) and its SNPs (12.5, 25, 50 µg mL^−1^), and 100 µL of the mixture was then added to the Vero cells seeded at 100 µL/well of 96-well microtiter plate at a density of 10^5^ cells/mL. Cells were incubated at 37 °C for 2 h with 5% CO_2_. Subsequently, cells were washed three times with phosphate buffer saline (PBS) and further incubated with 100 µL of DMEM containing 2% FBS. For post-infection exposure, 10^4.8^ and 10^3.7^ TCID_50_/mL for IBV and ILTV viruses, respectively were added to the Vero cells and incubated at 37 °C for 2 h in CO_2_ incubator (5% CO_2_). Thereafter, cells were washed three times and incubated with 100 µL of different concentrations *Cyperus rotundus* L. SNPs (12.5, 25, 50 µg mL^−1^). As controls for all experiments, mock-control and virus-infected control were included. All plates were incubated at 37 °C for 48 h in CO_2_ incubator and cells were fixed using paraformaldehyde (4%) at 20 °C for 10 min. The immunofluorescence staining of infected cells was carries out (see below).

#### Immunofluorescence staining of infected cells

The fixed cells were permeabilized with Triton X-100, 0.1% (Sigma–Aldrich GmbH, Steinheim, Germany). For the identification of virus-infected cells, the viral antigens were incubated with hyperimmune serum against ILTV and IBV containing 10% normal goat serum at 37 °C for 1 h, followed by washing with PBS for 3 times. Subsequently, the cells were incubation with fluorescein isothiocyanate (FITC)-labeled goat anti-rabbit IgG (Molecular Probes) for 1 h at 37 °C followed by washing for 3 times with PBS. Finally, the stained cells analyzed by fluorescence microscopy (Carl Zeiss, Germany) [58].

### Statistical evaluation

The null hypothesis was rejected, and the confidence level was at 99% that the parameters were significantly different found at *P* ≤ 0.01, using analysis of variance (ANOVA) one-way, followed by SMD. The statistical significance of the main results obtained from treating two viruses with both SNPs pre-treatment and post-treatment was compared to control [59].

## Conclusions

In conclusion, this is a novel research that studied for the first time the inhibitory effect of green synthesized silver *C. rotundus* nanoparticles as an antiviral agent against ILT and IB viruses and determine active compounds responsible for such antiviral activity. The HPLC profile of *C. rotundus* aqueous extract indicated that there are 14 phenolic and flavonoid compounds some of these has the antiviral efficacy like gallic acid, chlorogenic acid, methyl gallate, naringenin, kaempferol, quercetin and rutin. The results showed significant antiviral activity of *C. rotundus* SNPs in both pre-infection and post-infection exposures. However, the efficacy of antiviral activity was increased when the infected cells were post-treated with SNPs.

Green synthesized *C. rotundus* SNPs are potential potent antiviral agent against both IBV and ILTV. Further in vivo study is crucial to prove the antiviral effect of *C. rotundus* silver nanoparticles against both viruses in diseased chickens*.*

## Data Availability

All data are presented in tables and figures.

## Abbreviations

ATR-FT-IR: Attenuated total reflectance-Fourier transform-infrared spectrum
gB: Glycoprotein B
DLS: Dynamic light scattering
DMEM: Dulbecco’s modified Eagle’s medium
HPLC: High-performance liquid chromatography
HSV-1: Herpes simplex virus type 1
IB: Infectious bronchitis
IBV: Infectious bronchitis virus
ILTV: Infectious laryngotracheitis virus
MNTC: Maximum nontoxic concentration
RNS: Reactive nitrogen species
ROS: Reactive oxygen species
SPR: Surface plasmon resonance
SNPs: Silver nanoparticles
TEM: Transmission electron microscopy
